# Potential Impact of the OPTN Status Escalation Policy for Adult Heart Transplant Candidates With Durable LVADs

**DOI:** 10.1161/CIRCHEARTFAILURE.125.013854

**Published:** 2026-06-23

**Authors:** Daniel J. Ahn, Antony Attia, Toshihiro Nakayama, Nikhil Narang, Kiran K. Khush, William F. Parker, Kazunari Sasaki

**Affiliations:** Department of Surgery (D.J.A., A.A., T.N., K.S.), Stanford University, CA.; Department of Medicine (K.K.K.), Stanford University, CA.; Stanford Transplant Outcomes Research Center (D.J.A., A.A., T.N., K.S.), Stanford University, CA.; Stanford Cardiovascular Institute (D.J.A., K.K.K.), Stanford University, CA.; Department of Medicine, University of Chicago, IL (N.N., W.F.P.); Department of Public Health Sciences, University of Chicago, IL (W.F.P.); MacLean Center for Clinical Medical Ethics, University of Chicago, IL (W.F.P.).

**Keywords:** heart-assist devices, heart transplantation, policy

## Abstract

**BACKGROUND:**

The 2018 heart allocation policy change substantially lowered the priority of candidates supported with durable left ventricular assist devices (LVADs) for heart transplantation. To provide stable candidates supported by durable LVADs with a quicker path to transplantation before they suffer complications, the Organ Procurement and Transplantation Network (OPTN) approved a policy stipulating that stable patients supported by durable LVADs for 6 and 8 years will obtain statuses 3 and 2, respectively.

**METHODS:**

Using OPTN data, we identified all adult heart transplant candidates with a durable LVAD implanted between October 18, 2018 and May 31, 2025. We estimated the cumulative incidence of LVAD-related complications, treating transplantation and waitlist removal before experiencing complications as competing events. Furthermore, we assessed how the OPTN policy change would impact the status distribution of the waitlist.

**RESULTS:**

In our study cohort, 4967 adult patients who were listed for heart transplant received a durable LVAD. Transplant centers submitted 2879 justifications for status upgrades due to LVAD-related complications for 1812 (36.5%) patients. At 6 years after durable LVAD implantation, the cumulative incidence of complications and status upgrades was 42.1% (95% CI, 40.5%–43.8%), and that of transplantation was 36.0% (95% CI, 34.6%–37.6%). Of the 3779 patients who were not censored administratively, only 47 (1.2%) remained on the waitlist by 6 years after durable LVAD implantation. Had the 6- to 8-year OPTN policy change been implemented on June 1, 2025, only 4.7% of the waitlist would have changed statuses instantaneously.

**CONCLUSIONS:**

Almost all listed candidates with durable LVADs either experience a complication, status upgrade or are removed from the waitlist within 6 years of obtaining a durable LVAD. The upcoming OPTN policy is unlikely to prevent device complications before granting status upgrades and will likely impact a small percentage of candidates with durable LVADs.

**What is New?**

- This is the first study using complete national transplant registry data to project the potential impact of the Organ Procurement and Transplantation Network’s approved status escalation policy that upgrades durable left ventricular assist device (LVAD) candidates to status 2 and 3 after 6 and 8 years of device support, respectively.
- The total number of candidates supported by durable LVADs has decreased since October 2018, but the proportion of LVAD candidates supported for 6 years and longer has increased from 1.8% in October 2018 to 16.4% in June 2025.

**What Are the Clinical Implications?**

- The Organ Procurement and Transplantation Network time-based status escalation policy is unlikely to prevent durable LVAD complications, as the great majority of them occur before 6 years of durable LVAD support.
- Most candidates supported by durable LVADs are delisted long before they reach the 6-year timepoint of device support.
- The proposed 6- to 8-year policy for upgrading LVAD candidates would lead to minimal changes in the composition of the waitlist priority
- A more aggressive time-based escalation policy that upgrades candidates after shorter durations of support may reduce complication rates but would alter the composition of the waitlist significantly.

**See Editorial by Silvestry**

Since October 2018, the United States donor heart allocation system has rank ordered adult transplant candidates using 6 ordinal statuses.^1^ While patients who are clinically stable and supported by durable left ventricular assist devices (LVADs) used to have the second-highest waitlist priority before October 2018, they are now listed as status 4 as a default. In response to this drop in priority, transplant centers have significantly reduced their use of durable LVADs as a bridge to transplantation,^2–4^ shifting to temporary mechanical circulatory support devices such as intra-aortic balloon pumps, percutaneous endovascular ventricular assist devices, and extracorporeal membrane oxygenation.^5,6^

In response, the Organ Procurement and Transplantation Network (OPTN) recently approved a policy–that will be implemented in September 2026–supporting escalation in waitlist priority of durable LVAD candidates to statuses 3 and 2 after 6 and 8 years of durable LVAD support, respectively.^7^ While the concept of a time-served exception to afford durable LVAD candidates a greater chance at obtaining a transplant was well-received, many patients, advocacy groups, and transplant programs voiced concerns that waiting for 6 and 8 years is too long, with candidates potentially at high risk of experiencing device-related complications during this time.^7^ In this analysis, we aimed to determine whether the upcoming policy change would reduce the number of durable LVAD complications before status escalation and how it would impact the distribution of patients on the waitlist.

## Methods

### Data Source and Study Population

This study used data from the Scientific Registry of Transplant Recipients (SRTR). The SRTR files include data on all donors, waitlisted patients, and transplant recipients in the United States. The Health Resources and Services Administration provides oversight to the activities of the OPTN contractors. This study was exempted by the Stanford University Institutional Review Board. These data can be obtained upon request to the SRTR, and the complete code written to perform the analyses in this article is available in an online repository.^8^ We included all adult heart transplant candidates who were listed for transplantation on or before May 31, 2025 and had a durable LVAD implanted at any time between October 18, 2018 and May 31, 2025, regardless of whether durable LVAD implantation occurred before or after initial listing. Follow-up was available until June 30, 2025. This study followed STROBE guidelines (Strengthening the Reporting of Observational Studies in Epidemiology).

### Patient Characteristics

We collected relevant demographic and clinical data from patients at listing. Variables included sex, age at durable LVAD implantation, body mass index, race, blood type, primary diagnosis, and insurance type. We categorized body mass index into the following: underweight (<18.5 kg/m^2^), normal (18.5–24.9 kg/m^2^), overweight (25–29.9 kg/m^2^), and obese (≥30 kg/m^2^).

### Outcomes and Statistical Analysis

The primary outcomes of our study were rates of heart transplantation and complications or status upgrades related to durable LVAD support. As described in OPTN policy, specific LVAD-related complications such as device malfunctions, device infections, and mucosal bleeding meet standard listing criteria.^1^ However, similar to prior research,^9^ we considered any upgrade in status above status 4 among patients with durable LVADs to be due to a complication. For example, we reasoned that even though intra-aortic balloon pump and percutaneous endovascular ventricular assist device support (meeting status 2 criteria) or extracorporeal membrane oxygenation support (meeting status 1 criteria) are not explicitly categorized as complications, patients who are originally clinically stable with durable LVADs at status 4 but then require temporary mechanical circulatory support or any other type of support meeting elevated status criteria should be considered as having failure or complication of the durable LVAD. We categorized status upgrades and complications into 3 groups: (1) objective LVAD-related complications, (2) additional hemodynamic or mechanical circulatory support, and (3) exceptions. The only status upgrade criterion that we did not consider to be a complication was the 30 days of discretionary status 3× for durable LVAD patients. This specific indication for status 3 is not due to failure or complication of the device. It is rather intended to be a mechanism for providing elevated priority for a limited time while these patients remain clinically stable.^1^

We then estimated the cumulative incidence of experiencing a complication or status upgrade by 6 years after durable LVAD implantation. We treated heart transplantation and death or removal for deterioration before experiencing status upgrades and complications as competing events. We also stratified our analysis by type of durable LVAD placed and performed Fine-Gray analysis for all competing events. Because the SRTR does not collect information about when durable LVADs are explanted before delisting, we considered candidates to have had their LVADs explanted if and when they obtained status 5 or 6, because the lowest status that candidates with LVADs should have is status 4. Patients were censored if their durable LVAD was explanted (received status 5 or 6) before transplant, at the time of last follow-up, or administratively on June 30, 2025. We also identified all patients who received a transplant with a durable LVAD still in place and plotted the distribution of their statuses at transplant. We then obtained all status justifications submitted on behalf of patients who obtained a status higher than 4 at any time during listing after durable LVAD implantation.

### Temporal Trends in Durable LVAD Utilization and Policy Impact

To estimate the potential impact of various status escalation policies, we plotted the distribution of durable LVAD candidates at quarterly intervals from October 18, 2018, through June 1, 2025 and identified who would have been eligible for instantaneous status upgrades had the status escalation policies been implemented at each respective timepoint. The first policy scenario is phase 1 of the upcoming September 2026 OPTN policy, in which candidates who have had a durable LVAD for 6 and 8 years would receive status 3 and 2, respectively. The second scenario is phase 2 of the aforementioned policy, in which candidates with an LVAD for 5 and 7 years would get statuses 3 and 2, respectively. In reality, phase 2 of the policy would be implemented in early 2028.^7^ The final scenario is a hypothetical policy in which candidates with a durable LVAD for 2 and 4 years would receive status 3 and 2, respectively. We chose 2 years for status 3 due to recent evidence demonstrating that posttransplant survival decreases significantly among patients transplanted with a HeartMate 3 after 2 years of durable LVAD support.^10^ We then chose 4 years for status 2 simply by adding 2 years, as was done for both phases 1 and 2 of the upcoming policy. This specific analysis includes all patients with durable LVADs on the waitlist at each timepoint, including those with devices that were implanted before October 18, 2018.

To compare demographic and clinical data of patients stratified by type of durable LVAD, we performed descriptive statistics with χ^2^ tests for categorical variables and Wilcoxon rank-sum tests for continuous variables. All statistical tests were 2-sided, and we considered a *P* value of <0.05 to be significant. We performed all analyses with R (version 4.5.1).

### Sensitivity Analyses

To assess the robustness of our findings, we conducted 3 sensitivity analyses. First, to evaluate whether our results were driven primarily by exception-based status upgrades rather than objective LVAD-related complications, we repeated our cumulative incidence analysis, excluding all status upgrades justified by exceptions from the defined group of complications. Second, we reperformed our competing risks analysis using the listing date as the start time rather than the durable LVAD implantation. Third, to examine whether complication rates and outcomes changed over time, we stratified our cohort by LVAD implantation date into 2 time periods: October 18, 2018, through June 30, 2021 (early period) and October 1, 2021, through June 30, 2024 (late period). We estimated the cumulative incidence of complications and status upgrades at 3 years after LVAD implantation, treating transplantation before complications and waitlist removal as competing events. We performed Fine-Gray analysis to compare the incidence of varying competing outcomes between the 2 periods.

## Results

A total of 4967 adult heart transplant candidates at 123 US transplant centers had a durable LVAD placed between October 18, 2018 and May 31, 2025. Table 1 summarizes the demographic and clinical characteristics of all patients stratified by type of durable LVAD. Patients with the HeartMate 3 durable LVAD were more likely to be older at listing (56 versus 54 years; *P*<0.001) and to be male (80.9% versus 74.0%; *P*<0.001). There were no significant differences in blood type. Non-ischemic dilated cardiomyopathy was the most common diagnosis overall (48.3%). Four thousand two hundred ninety-eight (86.6%) of the durable LVADs were Abbott HeartMate 3, and 596 (12.0%) were Medtronic HeartWare ventricular assist device (HVAD). A full list of the durable LVAD types is provided in Table S1. A total of 33 patients were censored for LVAD explantation. There was significant variability in when durable LVADs were implanted with respect to initial listing, ranging from a median of 1120 days to 0 days before initial listing (Kruskal-Wallis *P*<0.001; Figure S1). Just 824 (16.6%) patients had their durable LVAD implanted on the day of or after initial listing. The median time from durable LVAD implantation to removal from the waitlist or administrative censoring was 1.77 years (interquartile range [IQR], 0.96–2.96 years). Follow-up for patients with HeartMate 3 LVADs (1.78 years; IQR, 0.99–2.96 years) was longer than that for patients with non-HeartMate 3 (1.68 years; IQR, 0.80–2.98 years; Kruskal-Wallis *P*=0.03).

**Table 1.**
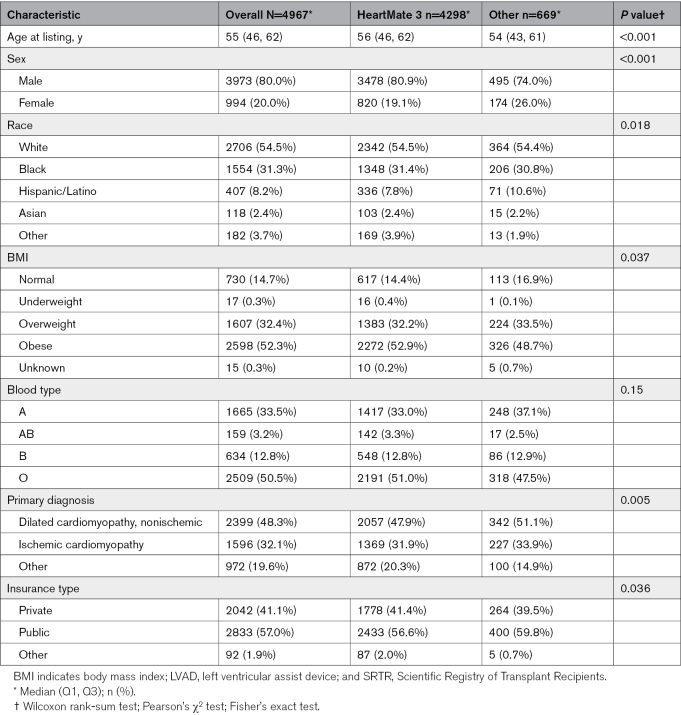
Demographic and Clinical Characteristics of Study Cohort, Stratified by Type of Durable LVAD (SRTR October 2018–June 2025)

### Complications and Status Upgrades

Transplant centers submitted a total of 2879 justification files for status upgrades/LVAD-related complications for 1812 patients (36.5%), with a median of 1 file (IQR, 1–2) submission per patient. These justifications comprised 3 groups: objective LVAD-related complications (n=1378; 47.9%), additional hemodynamic or mechanical support (n=191; 6.6%), and exception-related justifications (n=1310; 45.5%; Table 2). Among objective complications, device infections were the most common (64.8%), followed by device malfunction (10.2%) and aortic insufficiency (7.0%). Exception-related justifications accounted for the majority of status 1 (70.8%) and status 2 (72.2%) upgrades, while device infections accounted for the majority of status 3 upgrades (59.7%). The frequency of justifications submitted for objective LVAD-related complications and exceptions differed significantly by durable LVAD type (both *P*<0.001) but not for additional hemodynamic or mechanical support (*P*=0.41). Notably, candidates with the HVAD had substantially higher rates of device malfunction (22.6% versus 7.6%) and pump thrombosis compared with HeartMate 3 (12.4% versus 3.2%). Overall, 2864 (99.5%) justification files were submitted within 6 years of durable LVAD implantation, with a median time to first submission of 603 days (IQR, 296–1069.5 days).

**Table 2.**
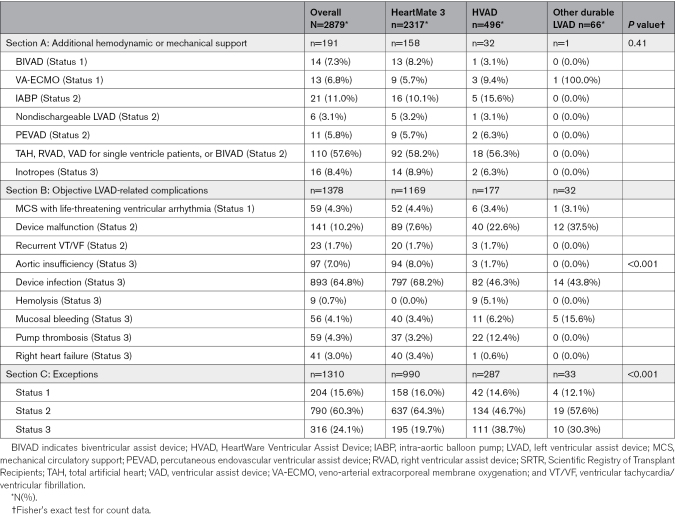
Durable LVAD Complications or Status Upgrades During Listing, Stratified by Type of Device (SRTR October 2018–June 2025)

**Figure 1.**
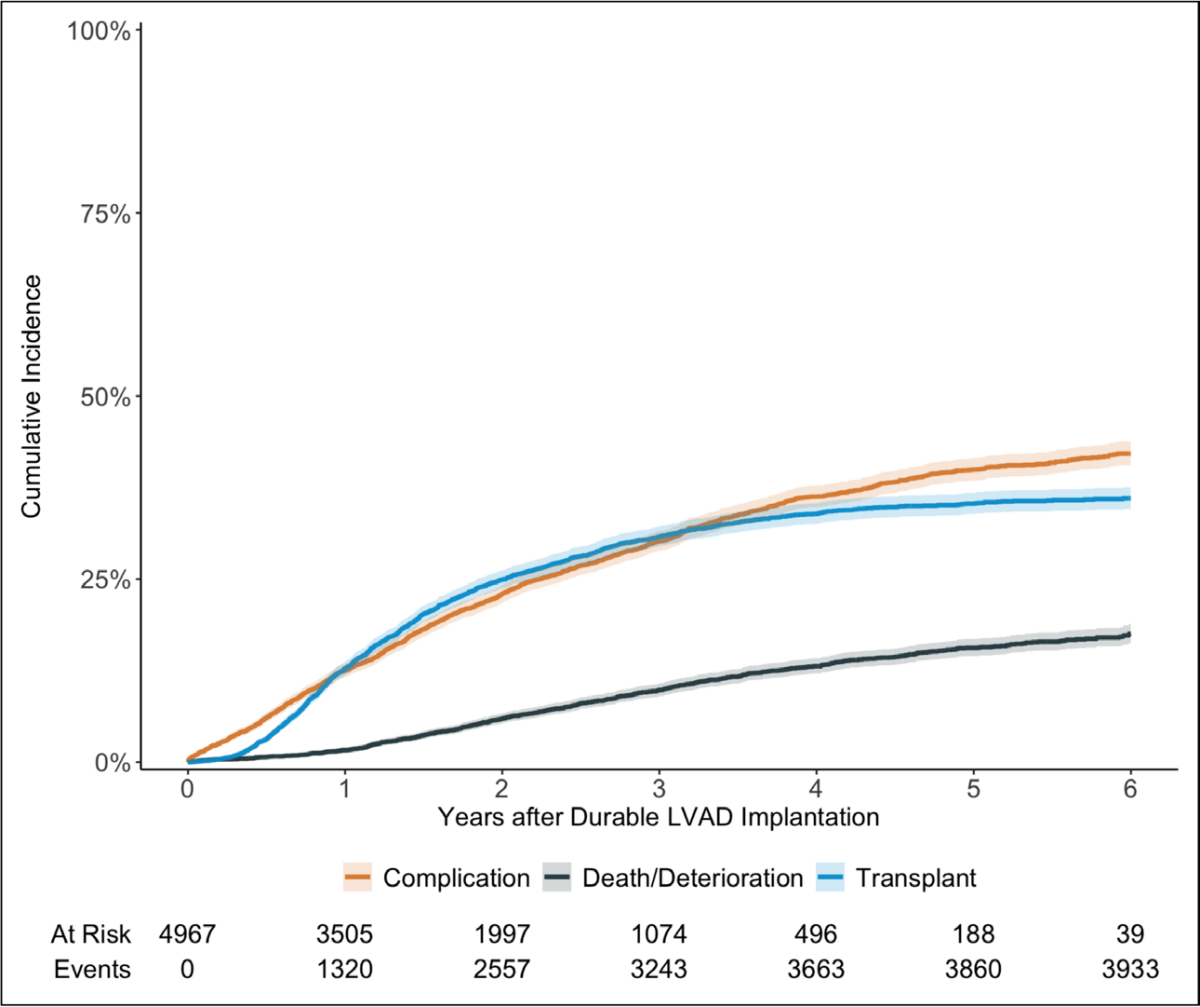
**Cumulative incidence of complications or status upgrades among 4967 candidates with durable left ventricular assist devices (LVADs).** Transplantation and death or removal for clinical deterioration before experiencing complications and status upgrades are treated as competing events. The cumulative incidence of complications at 6 years was 42.1% (95% CI, 40.5%–43.8%). For transplant and death or deterioration, the incidences were 36.0% (95% CI, 34.6%–37.6%) and 17.5% (95% CI, 16.2%–18.9%), respectively.

The cumulative incidence of experiencing any complication or status upgrade (other than status 3 for discretionary LVAD time) at 6 years was 42.1% (95% CI, 40.5%–43.8%), which was higher than that of transplantation at status 4 or status 3 for discretionary LVAD time (cumulative incidence, 36.0% [95% CI, 34.6%–37.6%]; Figure 1). The estimated cumulative incidence of death or removal for clinical deterioration was 17.5% (95% CI, 16.2%–18.9%). Patients with HeartMate 3 devices were significantly less likely to experience a complication or status upgrade (39.6% [95% CI, 37.9%–41.4%] versus 52.7% [95% CI, 49.0%–51.6%], Fine-Gray *P*<0.001) than those with non-HeartMate 3 durable LVADs (Figure S2). One thousand one hundred eighty-eight patients (23.9%) of the cohort were censored administratively before they reached 6 years of durable LVAD support. Of the remaining 3779 (76.1%) patients, only 47 (1.2%) remained on the waitlist by 6 years after durable LVAD implantation.

### Heart Transplants With Durable LVADs

**Figure 2.**
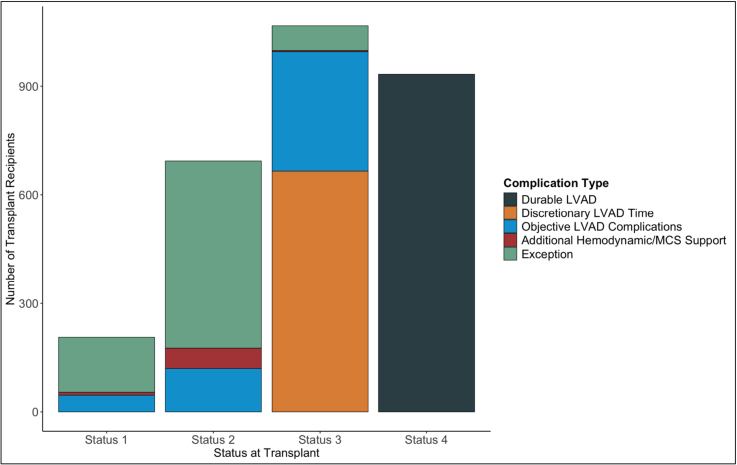
**Distribution of status at the time of heart transplantation.** Two thousand eight hundred and ninety-nine patients with durable left ventricular assist devices (LVADs) obtained heart transplants, and the statuses are further stratified by complication type, including objective LVAD-related complications, additional hemodynamic or mechanical circulatory support, and exceptions. Among 1067 patients with status 3 at transplant, 665 (62.3%) had status 3 by exercising their 30 days of discretionary durable LVAD time.

Two thousand eight hundred and ninety-nine (58.4%) patients received a heart transplant with a durable LVAD in place, and the distribution of statuses they had at transplant is available in Figure 2. Two hundred and six (7.1%) were status 1, 693 (23.9%) were status 2, 1067 (36.8%) were status 3, and 933 (32.2%) were status 4. Six hundred and sixty-five (62.3%) of the patients with status 3 were exercising their 30 days of discretionary LVAD time. Exceptions accounted for the majority of transplant recipients with status 1 (73.8%) and status 2 (74.6%). Of the 865 patients removed from the waitlist without transplantation, 288 (33.3%) died or were removed for clinical deterioration, 22 (2.5%) were removed for clinical recovery, and 555 (64.2%) were removed for other reasons.

### Impact of Time-Based Status Escalation Policies by the OPTN

**Figure 3.**
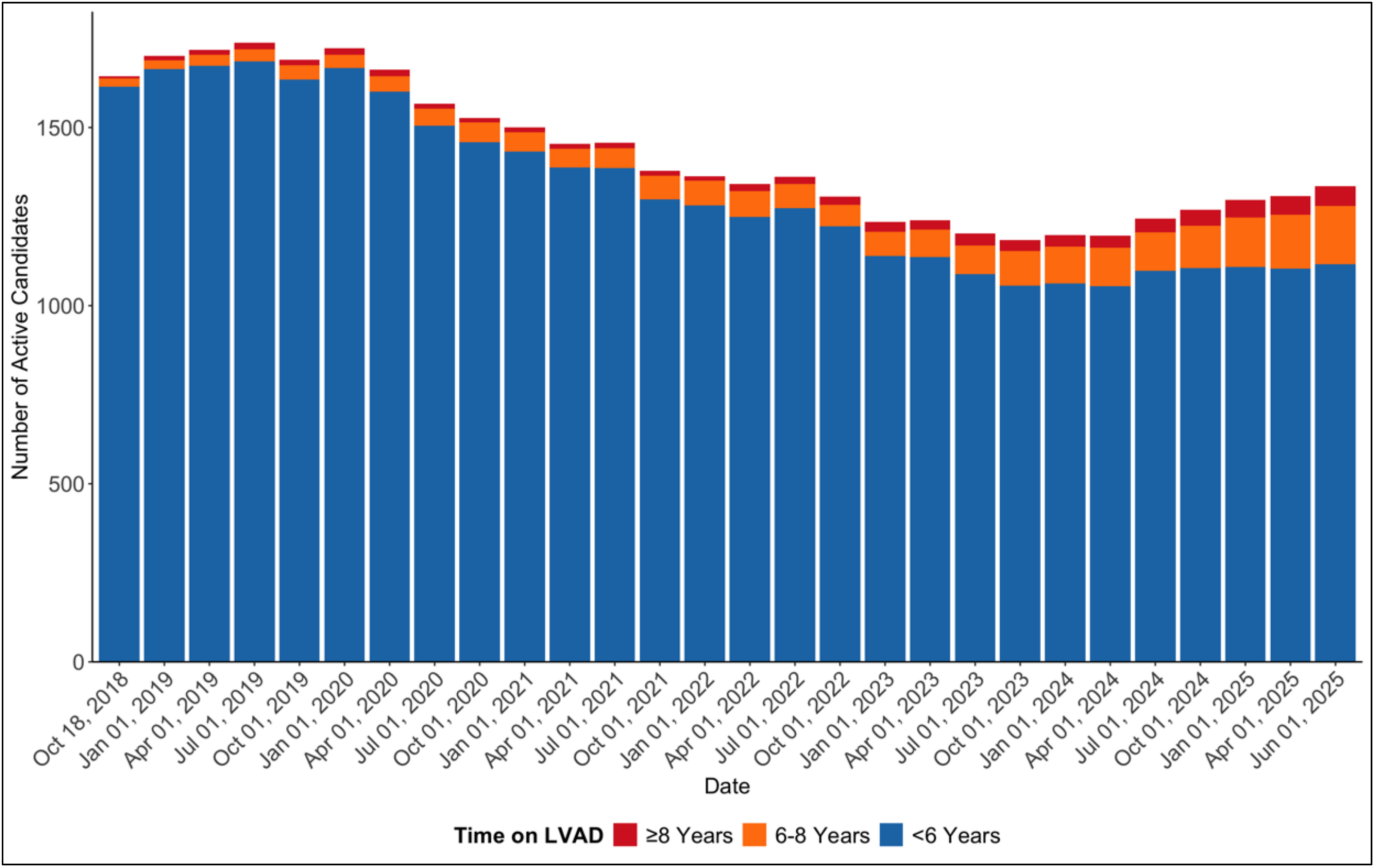
**Total number of candidates supported by durable left ventricular assist devices (LVADs) at specified timepoints between October 18, 2018 and June 1, 2025.** Bars are stratified by duration of durable LVAD support at each timepoint, including <6 years, 6 to 8 years, and ≥8 years. The total number of candidates supported by durable LVADs has decreased, but the proportion of candidates with long durations of device support ≥6 years has increased.

The total number of candidates supported by durable LVADs has decreased, starting with 1644 on October 18, 2018, to 1335 on June 1, 2025 (Figure 3). In contrast, the number of candidates supported by durable LVADs for 6 years and longer has increased monotonically, from 30 out of 1644 (1.8%) candidates with LVADs on October 18, 2018, to 219 out of 1335 (16.4%) on June 1, 2025.

**Figure 4.**
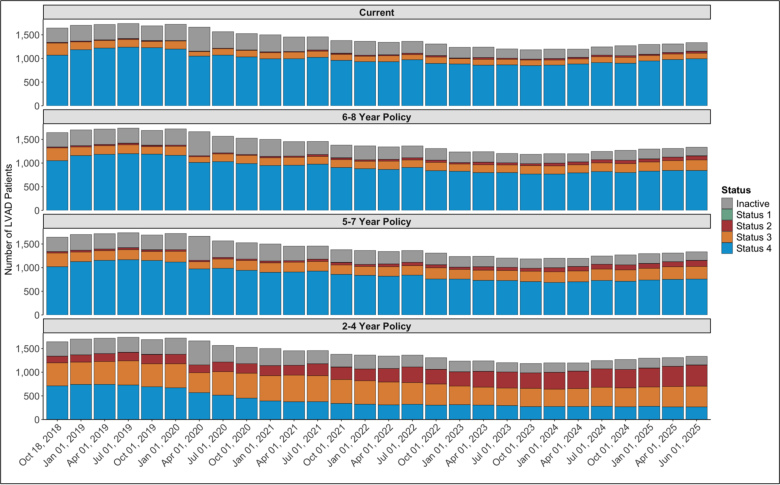
**Status distribution of candidates supported by durable left ventricular assist devices (LVADs) under varying status escalation policies.** The first panel represents the actual status distribution of candidates supported by durable LVADs at each of the quarterly timepoints listed between October 18, 2018 and June 1, 2025. The next 3 panels represent what the status distribution would have looked like had the 6- to 8-year policy, 5- to 7-year policy, and a hypothetical 2- to 4-year policy been implemented at each of the same timepoints, respectively.

Under both phases of the upcoming OPTN status escalation policy, the number of additional candidates supported by durable LVADs who would have been upgraded instantaneously had the policy been implemented at any of the specified quarterly timepoints between October 18, 2018, and June 1, 2025 is small (Figure 4; Table S2). Under phase 1, the median number of candidates who would have been instantly added to status 3 and 2, respectively, is 41 (IQR, 27.5–59) and 15.5 (IQR, 12–24.2). Under phase 2, the median number of candidates upgraded to status 3 and 2 is 75 (IQR, 61.2–105) and 31.5 (IQR, 21–46.8), respectively. Had phase 1 or phase 2 of the OPTN policy been implemented on June 1, 2025, the proportion of the entire waitlist that would have been upgraded to either status 3 or 2 is 194 out of 4158 (4.7%) and 301 out of 4158 (7.2%), respectively (Figure S3; Table S3).

In contrast, a hypothetical policy granting status 3 after 2 years and status 2 after 4 years would have had substantial impact throughout the entire study period, with a median of 322 (IQR, 277–358) patients added to status 3 and 252 (IQR, 192–319) to status 2 on any of the given dates (Figure 4; Table S2). On June 1, 2025, the proportion of the entire waitlist that would have been upgraded under this policy is 917 out of 4158 (22.1%) (Figure S3; Table S3).

### Sensitivity Analyses

First, in a sensitivity analysis in which we did not count listings with status exceptions as LVAD-related complications, we found that the cumulative incidence of complications or status upgrades at 6 years was 26.3% (95% CI, 25.0%–27.8%), lower than that in our primary analysis in which we considered status upgrades associated with exceptions as complications (Figure S4). Second, when designating the listing date as the start time rather than the LVAD implantation date, we found that the incidence of complications at 6 years was similar at 40.9% (95% CI, 39.3%–42.5%; Figure S5). The median follow-up time after initial listing was 0.53 years (IQR, 0.12–0.98 years). Of note, 729 out of 4967 (14.7%) patients were initially listed with a complication/status upgrade. Third, we found that candidates with durable LVADs between October 2021 and June 2024 (33.9% [95% CI, 31.3%–36.7%]) had a significantly higher rate of complications than between October 2018 and June 2021 (26.9% [95% CI, 25.1%–28.9%]; Fine-Gray *P*<0.001; Figure S6).

## Discussion

In this registry-based study of adult patients supported by durable LVADs awaiting heart transplantation in the US, we report the following findings: (1) the cumulative incidence of complications or status upgrades was >40% by 6 years after durable LVAD implantation, (2) nearly 100% of patients in the study cohort experienced a complication or were removed from the waitlist by 6 years after durable LVAD implantation, and (3) the upcoming OPTN policy change would likely have minimal impact on the overall composition of the waitlist.

Despite advances in durable LVAD technology that have conferred lower morbidity and mortality,^11–14^ our results support prior literature reporting that the number of patients listed with durable LVADs has decreased significantly.^4,15^ Only if patients develop device-related complications, such as refractory infections and pump malfunctions, can they be upgraded from status 4 to a higher status.^1^ As a result, transplant centers have moved away from durable LVADs while simultaneously increasing use of temporary mechanical circulatory support as a bridge-to-transplant strategy. In particular, rates of intra-aortic balloon pump utilization rose more than 3-fold after the allocation policy change in 2018.^16^ Indeed, while the number of LVAD candidates has decreased, the subgroup of candidates supported by durable LVADs for 6 years and longer has accrued. This is most likely because, before October 2018, clinically stable LVAD candidates had the second-highest waitlist priority, enabling transplantation much earlier than 6 years. Furthermore, as the new allocation system has matured, we observed significant time-dependent effects. In our sensitivity analysis comparing early (October 2018–June 2021) versus late (October 2021–June 2024) periods, the rate of complications and death/deterioration increased significantly, likely reflecting increasingly longer waitlist durations for patients with durable LVADs and thus increased risk of complications. While the OPTN has recognized the dwindling numbers of patients with durable LVADs awaiting heart transplant and will implement a time-served policy in September 2026 to help reduce the incidence of complications, our results show that 6 and 8 years may be too long to wait for a status upgrade. Notably, the median time from durable LVAD implantation to first documented complication or status upgrade was <2 years, underscoring that the clinically meaningful risk window occurs years before the 6-year policy threshold for the patients who do experience adverse events.

The OPTN Heart Committee wrote this policy based on an analysis of the adult heart waitlist as of April 30, 2024 and aimed to avoid a large influx of high priority listings that may negatively impact the waitlist survival of other candidates already listed as status 2 and 3.^7,17^ Thus, the committee decided on 6 and 8 years because these timeframes limited the total number of candidates with durable LVADs switching instantaneously to statuses 3 and 2, compared with other potential proposals.^7^ However, this analysis did not account for the fact that nearly all waitlist removals and complications leading to status upgrades occurred before the 6- to 8-year policy would provide any benefit in our study. The policy is intended to benefit candidates who are stable with durable LVADs and provide increased priority before complications occur, but <2% of patients with durable LVADs in our cohort after October 2018 were still on the waitlist by 6 years. The cumulative incidence of complications or status upgrades appears to plateau after 3 to 4 years of durable LVAD support, suggesting that the window during which time-based escalation may plausibly alter outcomes is concentrated several years before the 6-year threshold.

Furthermore, our temporal simulation analysis demonstrates that the total number of people who would have been eligible for status upgrades under either phase of the OPTN policy is low. The number of eligible candidates has increased over time, but this likely represents a transient cohort effect. Patients currently listed with 6+ years of support received their durable LVADs before or shortly after the 2018 allocation policy change, when implant rates were substantially higher. Given extensive literature reporting that the number of durable LVADs entering the waitlist has decreased substantially after 2018,^4,18,19^ the pipeline of patients who will reach 6 to 8 years in the near future will likely start to decline as time progresses. However, even with the increase in the number of patients eligible for status upgrades from 2018 to mid-2025, the percentage of the waitlist who would have been upgraded to status 3 or 2 had the 6 to 8-year policy been implemented on June 1, 2025 is still <5%.

Moreover, recent findings have shown that posttransplant survival among patients with durable LVADs is significantly worse when they are transplanted with complications meeting criteria for status 3 and 2 compared with when they are clinically stable with status 4.^9^ In the same vein, posttransplant survival may be worse following 2 years of durable LVAD support among patients with HeartMate 3 devices.^10^ Given the median time to complication was <2 years in our study, it is unlikely for the OPTN policy to alter posttransplant survival outcomes among candidates with durable LVADs. It may potentially make more sense for a more aggressive 2 to 4-year policy for patients with durable LVADs based on this data. However, when we simulated this policy temporarily, the potential impact was dramatic. On June 1, 2025 >900 candidates out of 4158 candidates would have been upgraded to status 3 and 2, which aligns with the OPTN Heart Committee’s concerns that a time-based policy that is too aggressive may overwhelm the system too quickly. Additionally, while the 2- to 4-year policy may certainly be more effective in preventing complications, only about 40% of candidates in our cohort ever experienced a complication by 6 years. Implementing such a short timeframe would grant upgrades to many LVAD candidates who never experience any complication, allowing them to compete equally with and potentially displace acutely ill patients already in status 2 and 3, which may be unfair.

Overall, while the 6- to 8-year policy may be too long to help patients with durable LVADs to obtain higher waitlist priority before developing complications, opting for a more aggressive policy that upgrades patients after shorter durations of LVAD support may lead to major alterations in waitlist composition that could negatively affect overall waitlist survival. As a result, a uniform time-based escalation may be misaligned with the heterogeneity of outcomes of candidates supported by durable LVADs. Instead of automatic upgrades based solely on device duration, a more targeted approach might identify subgroups at highest risk for complications or clinical deterioration who would benefit most from earlier upgrades. One potential approach is to base prioritization on when the survival benefit afforded by heart transplantation is maximal. Unlike medical urgency, which reflects immediate risk, survival benefit accounts for both pretransplant risk and posttransplant outcomes. Given that both pre- and posttransplant mortality is affected significantly by device complications, there may be an optimal window where transplantation yields maximum life-years, after sufficient device duration to justify donor heart utilization but before complications develop that compromise outcomes after transplant. Such a framework may better align policy with the goal of optimizing both pre- and posttransplant outcomes rather than attempting to equate the medical urgency of fundamentally different clinical scenarios.

### Limitations

There are several potential limitations to our study. First, we defined complications and status upgrades based on the status justification files submitted by transplant centers. Because the majority of candidates in our cohort obtained durable LVADs before listing, there may be complications missed during the period between LVAD implantation and initial listing, potentially exaggerating the length of time between LVAD implantation and documented complications. Furthermore, we did not include temporary inactivations as complications despite evidence that inactivations are typically related to poor functional or clinical status,^20^ which could be indicative of complications related to durable LVAD support. As a result, we may have underestimated how many complications actually occurred in this population.

Second, we defined all upgrades above status 4, except status 3, for discretionary LVAD time as complications, even if they are not explicitly categorized as LVAD-related complications in OPTN policy. However, we argue that any adverse clinical event that causes patients with durable LVADs to meet criteria for increased waitlist priority above what is afforded by status 4 should be considered a complication. Exceptions accounted for a significant portion of the status upgrades within this cohort. Indeed, in our sensitivity analysis excluding exceptions, such as LVAD-related complications, the cumulative incidence of status upgrades/complications decreased significantly. Exceptions are associated with lower medical urgency in general,^21,22^ but OPTN policy^1^ states that patients with approved exceptions in statuses 1 to 3 must be admitted to the hospitals where they are listed. As a result, we argue that exceptions should be included because inpatient admissions with a durable LVAD should be considered as adverse events.

Third, we only included patients who had durable LVADs placed after October 2018 in our cohort, which may have limited our study population. However, we restricted our cohort because we wanted to study the status upgrade and complication rate of recently placed durable LVADs, particularly the HeartMate 3, to maintain clinical relevance.

Lastly, our temporal simulation analysis reflects static reclassification of candidates at specified timepoints and does not model downstream effects on organ offers, match run dynamics, or regional sharing patterns. The true waitlist impact of the time-based policies had they been implemented previously may therefore differ from what our analysis suggests.

### Conclusions

The cumulative incidence of status upgrades and complications among heart transplant candidates with durable LVADs was >42% by 6 years after implantation. Furthermore, nearly every patient in our cohort experienced a complication or was removed from the waitlist within 6 years after durable LVAD implantation. The OPTN’s September 2026 policy change to escalate patients to statuses 3 and 2 after 6 and 8 years of durable LVAD support, respectively, is unlikely to prevent durable LVAD complications or materially change the composition of the waitlist for the majority of candidates.

## Article Information

### Disclosures

Dr Narang was a speaker for Boehringer Ingelheim and AstraZeneca and provided consulting services for BridgeBio. The other authors report no conflicts. The interpretation and reporting of these data are the responsibility of the authors and in no way should be seen as an official policy of or interpretation by the Organ Procurement and Transplantation Network or the US government.

### Supplemental Material

Tables S1–S3

Figures S1–S6

## Supplementary Material


