# Supplementary material for: Potential Impact of the OPTN Status Escalation Policy for Adult Heart Transplant Candidates With Durable LVADs
Source: Circ Heart Fail. 2026 Jun 23;19(7):e013854. doi: 10.1161/CIRCHEARTFAILURE.125.013854 (PMC13293278; doi:10.1161/CIRCHEARTFAILURE.125.013854)
Supplement: Supplementary file 1 [file hhf-19-e013854-s001.pdf]

SUPPLEMENTAL MATERIAL

Figures S1-S6
Table S1-S3

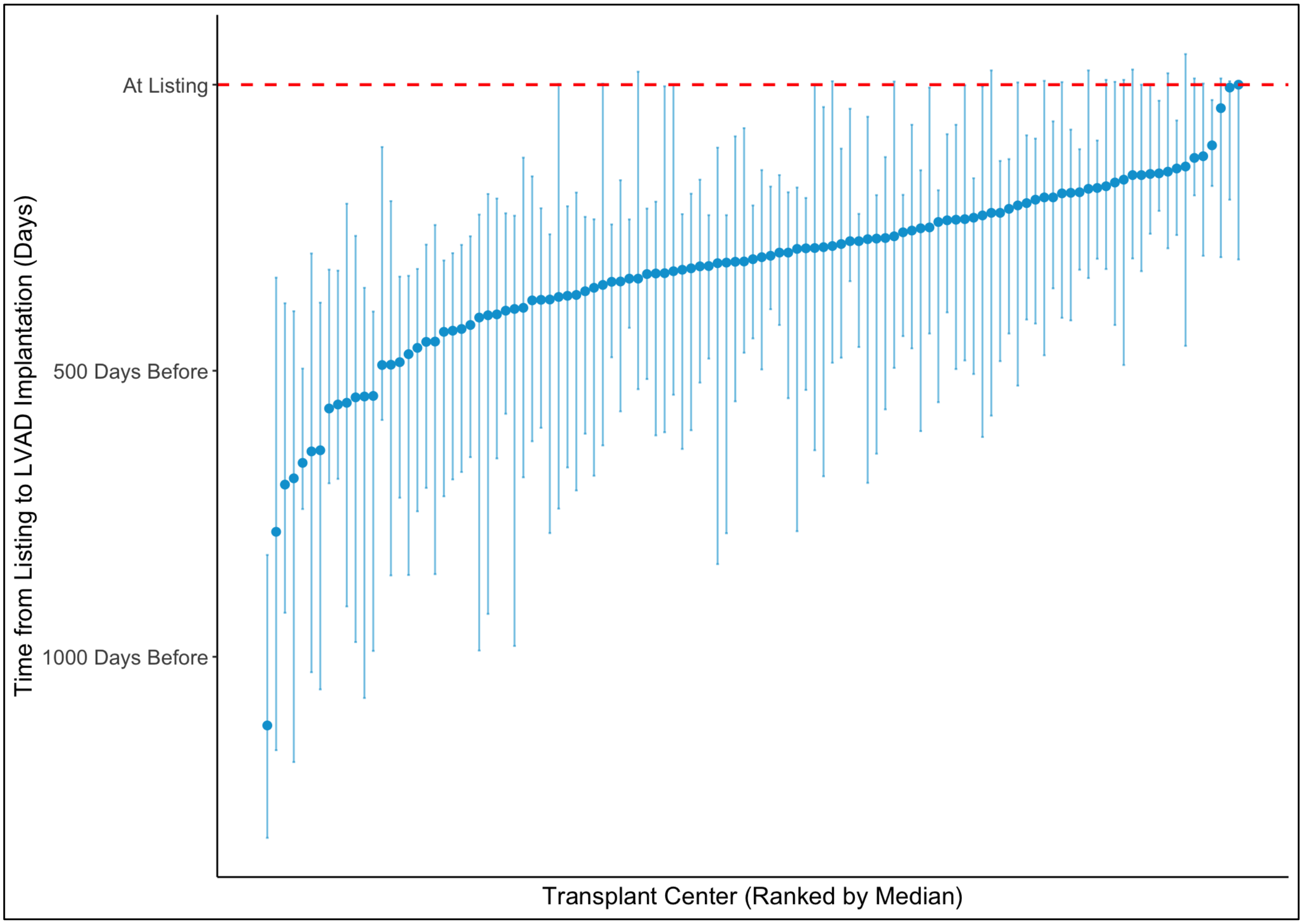

Figure S1: Center-level variation in timing of durable LVAD implantation relative to initial listing. The median timing and interquartile range (IQR) for each center is represented by dots and vertical lines, respectively. To calculate IQR for each center, we removed centers with <5 durable LVAD candidates. In total, 12 centers with 25 patients were removed from this figure, leaving a total of 4,942 out of 4,967 candidates at 111 out of 123 transplant centers. The median timing of implantation ranged from 1120 days to 0 days prior to initial listing (Kruskal-Wallis $p < 0.001$).

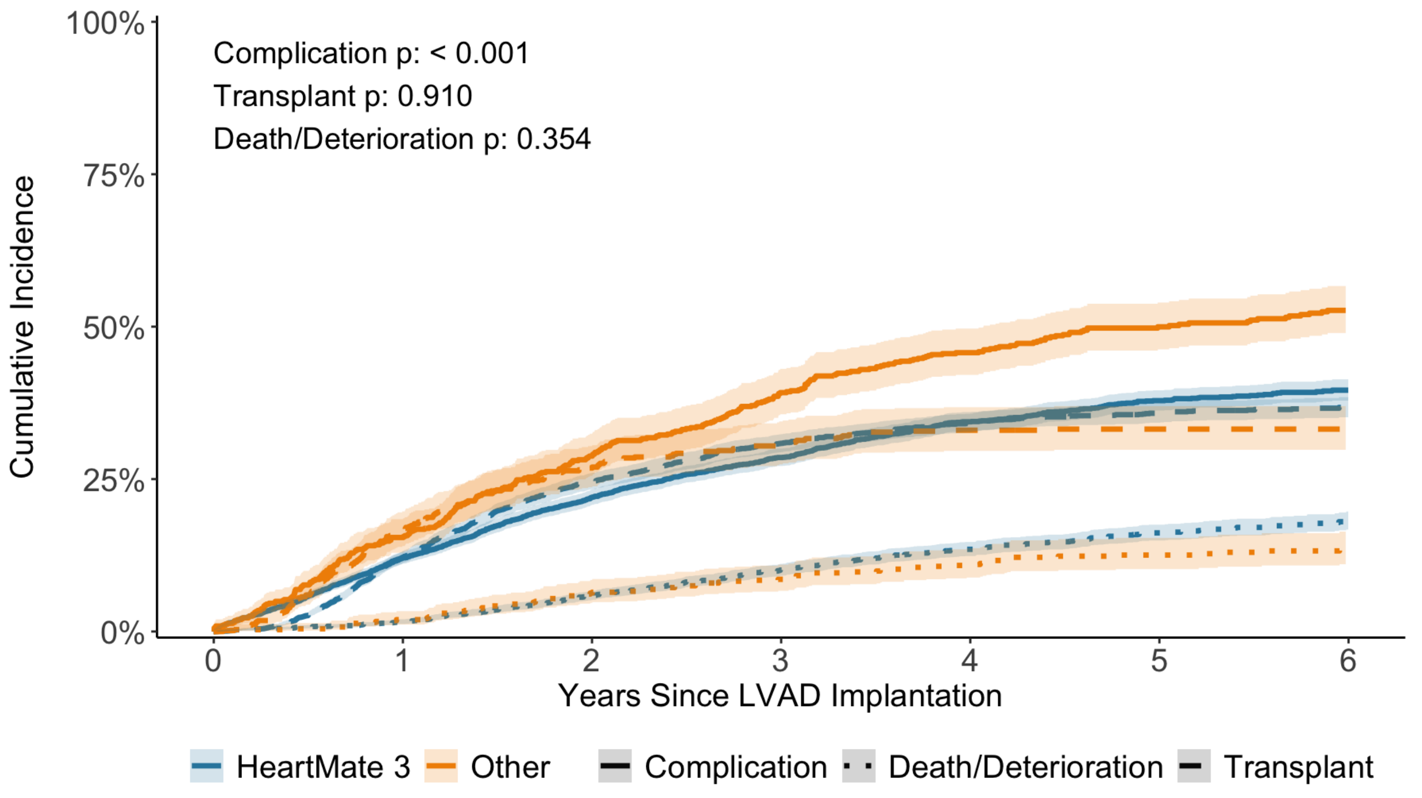

	0	1	2	3	4	5	6
**HeartMate 3**							
At Risk	4299	3065	1743	932	431	166	37
Events	0	1093	2144	2720	3066	3229	3285
**Other**							
At Risk	668	440	254	142	65	22	2
Events	0	227	413	523	597	631	648

Figure S2: Cumulative incidence of complications or status upgrades, stratified by durable LVAD type. Transplantation and waitlist removal prior to complications or status upgrades are competing events. Fine-Gray results for all competing events are in the top left of the figure. The numbers of patients at risk every year after durable LVAD implantation are shown at the bottom of the figure legend.

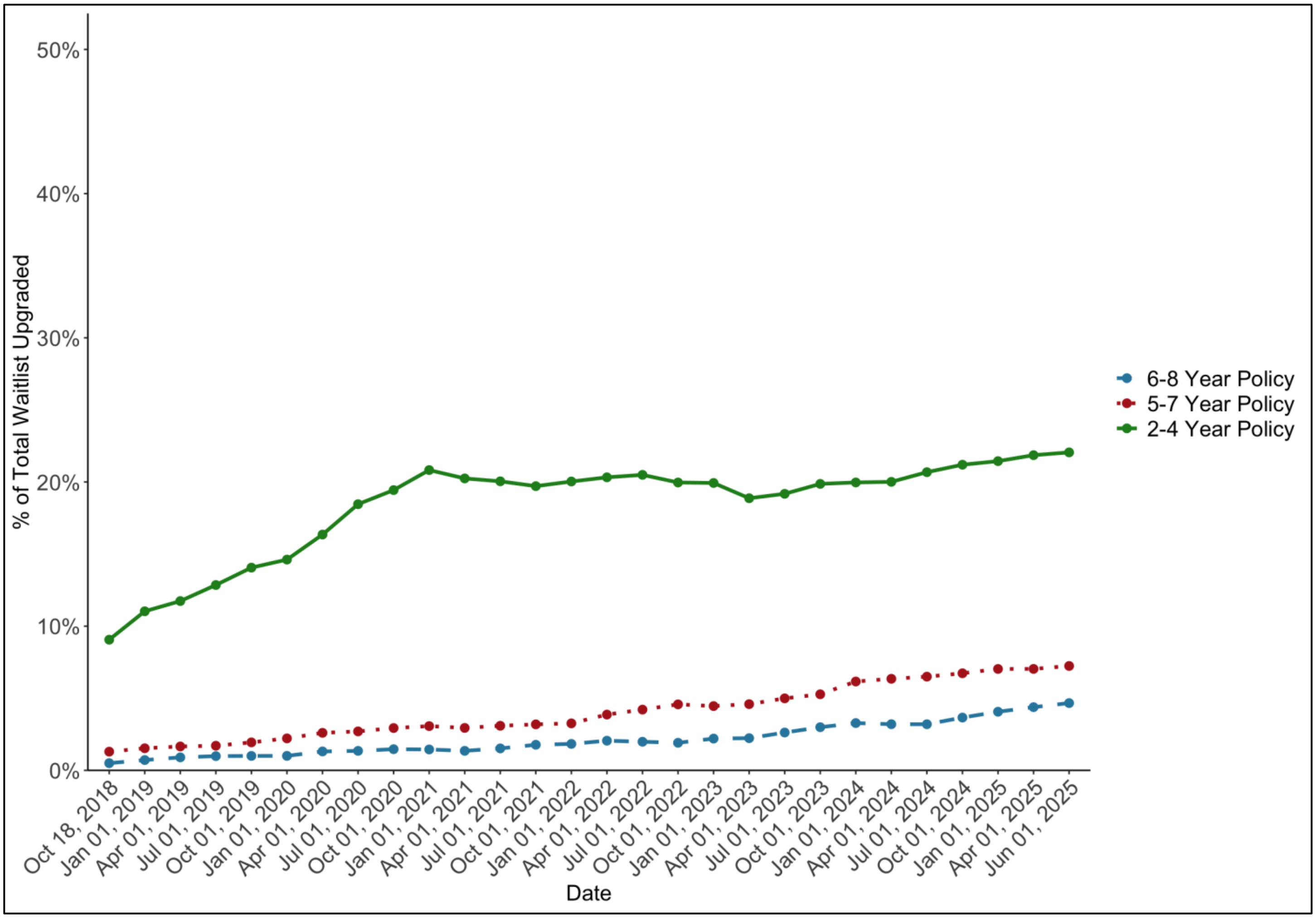

Figure S3: Total proportion of the waitlist that would have been upgraded had status escalation policies been implemented on specified dates from October 18, 2018 through June 1, 2025. The three status escalation policies represented are phase 1 of the approved OPTN policy (6- to 8-years), phase 2 (5- to 7-years), and a hypothetical 2- to 4-year policy.

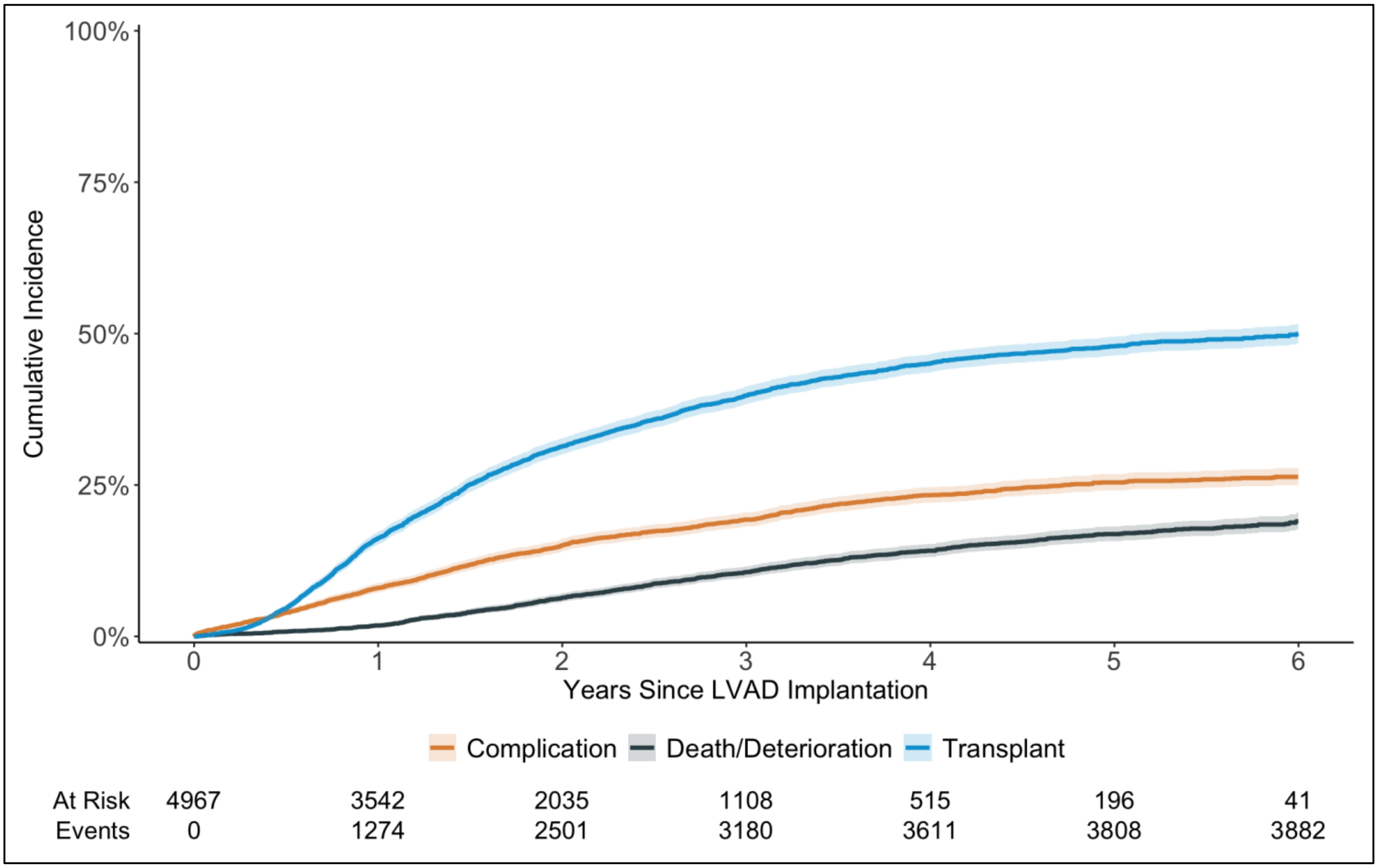

Figure S4: Cumulative incidence of complications or status upgrades excluding exceptions at 6 years after durable LVAD implantation, treating transplant and waitlist removal before complications/status upgrades as competing events. The cumulative incidence of complications or status upgrades at 6 years was 26.3% (95% CI [25.0%, 27.8%]), lower than that in our primary analysis (42.1% [95% CI (40.5%, 43.8%)]). The cumulative incidence of transplantation without complications was 49.9% (95% CI [48.3%, 51.6%]), and the cumulative incidence of death or deterioration before experiencing complications was 19.0% (95% CI [17.7%, 20.4%]).

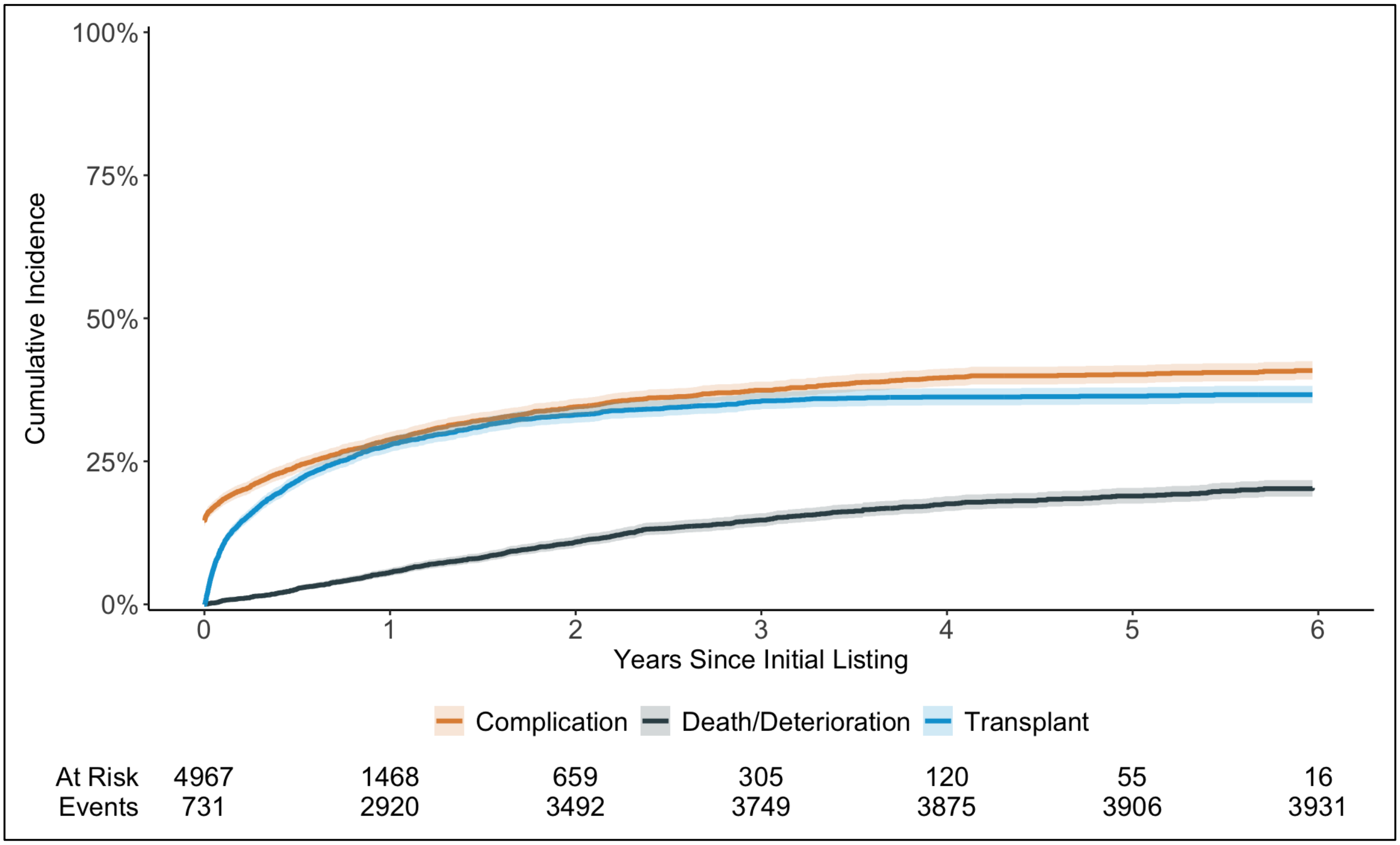

Figure S5: Cumulative incidence of complications or status upgrades up to 6 years after initial listing, treating transplant and waitlist removal before complications/status upgrades as competing events. The cumulative incidence of complications or status upgrades at 6 years was 40.9% (95% CI [39.3%, 42.5%]). The cumulative incidence of transplantation without complications was 36.6% (95% CI [35.2%, 38.2%]), and the cumulative incidence of death or deterioration before experiencing complications was 20.4% (95% CI [18.9%, 21.9%]).

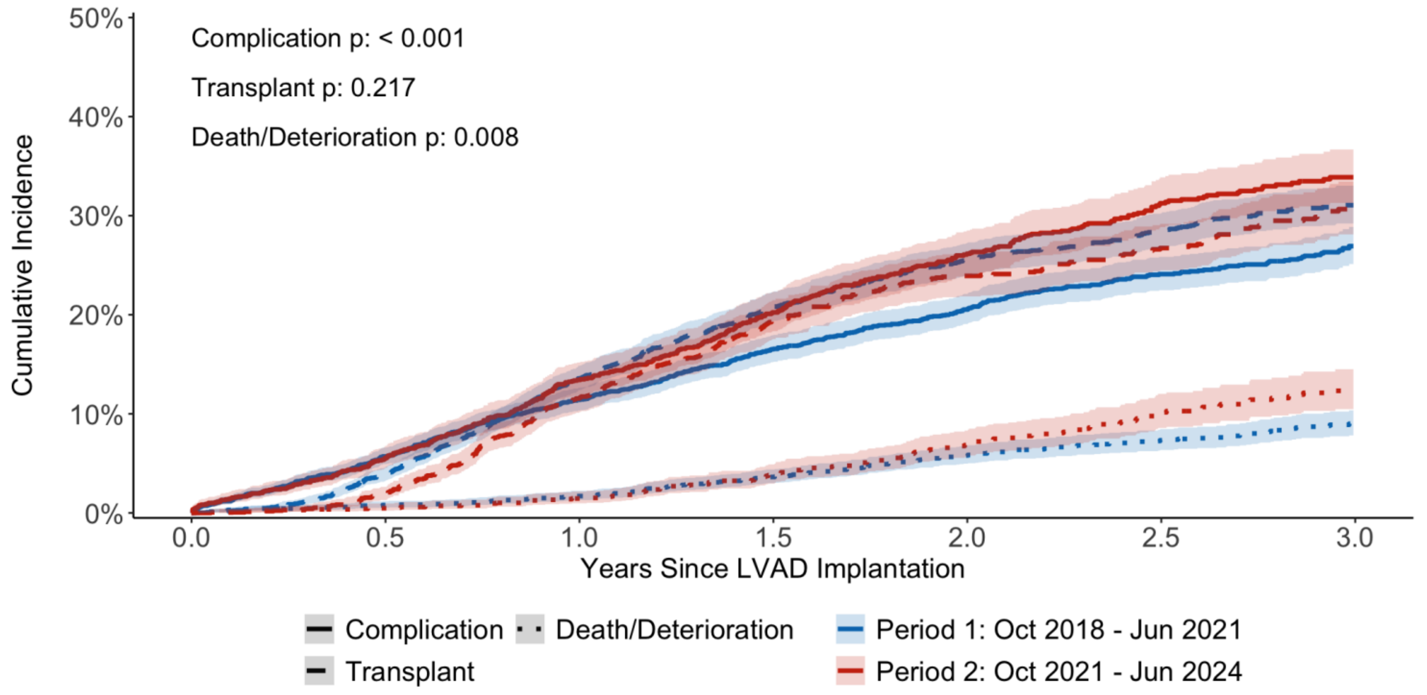

	0.0	0.5	1.0	1.5	2.0	2.5	3.0
**Period 1: Oct 2018 - Jun 2021**							
At Risk	2999	2682	2185	1499	966	585	315
Events	0	311	799	1190	1443	1584	1670
**Period 2: Oct 2021 - Jun 2024**							
At Risk	1682	1538	1163	784	478	254	103
Events	0	132	437	689	860	965	1021

Figure S6: Cumulative incidence of complications or status upgrades at 3 years after durable LVAD implantation, stratified by era. Transplant and waitlist removal before complications or status upgrades are treated as competing events. The cumulative incidence of complications was 26.9% (95% CI [25.1%, 28.9%]) in Period 1 (Oct 2018 - Jun 2021) compared to 33.9% (95% CI [31.3%, 36.7%]) in Period 2 (Oct 2021 - Jun 2024) (Fine-Gray $p < 0.001$). The cumulative incidence of transplantation was 31.1% (95% CI [29.2%, 33.0%]) in Period 1 and 30.7% (95% CI [28.1%, 33.4%]) in Period 2 (Fine-Gray $p = 0.217$). The cumulative incidence of death or deterioration was 9.0% (95% CI [7.8%, 10.4%]) in Period 1 and 12.3% (95% CI [10.5%, 14.5%]) in Period 2 (Fine-Gray $p = 0.008$)

Table S1: Brands of Durable LVADs among Waitlisted Candidates, October 2018-June 2025

	**N = 4,967***
HeartMate 3	4,298 (86.6%)
Heartware HVAD	596 (12.0%)
HeartMate II	56 (1.1%)
Evaheart	10 (0.2%)
Worldheart Levacor	2 (0.0%)
NuPulseCV iVAS	1 (0.0%)
Other	4 (0.0%)

* n (%)

Table S2: Number of Durable LVAD Candidates with Status 2 and 3 under Status Escalation Policies

Date	Total Waitlist	6-8 Year Policy	5-7 Year Policy	2-4 Year Policy

		N	%	N	%	N	%
Oct 18, 2018	5010	25	0.50%	65	1.30%	454	9.06%
Jan 01, 2019	4895	35	0.72%	75	1.53%	540	11.03%
Apr 01, 2019	4889	44	0.90%	81	1.66%	574	11.74%
Jul 01, 2019	4862	48	0.99%	83	1.71%	625	12.85%
Oct 01, 2019	4794	48	1.00%	93	1.94%	674	14.06%
Jan 01, 2020	4782	48	1.00%	106	2.22%	699	14.62%
Apr 01, 2020	4574	60	1.31%	119	2.60%	748	16.35%
Jul 01, 2020	4220	57	1.35%	114	2.70%	779	18.46%
Oct 01, 2020	4157	61	1.47%	122	2.93%	808	19.44%
Jan 01, 2021	4139	60	1.45%	127	3.07%	862	20.83%
Apr 01, 2021	4213	57	1.35%	124	2.94%	853	20.25%
Jul 01, 2021	4270	65	1.52%	132	3.09%	856	20.05%
Oct 01, 2021	4170	74	1.77%	133	3.19%	822	19.71%
Jan 01, 2022	4143	76	1.83%	135	3.26%	830	20.03%
Apr 01, 2022	4084	84	2.06%	158	3.87%	830	20.32%
Jul 01, 2022	4084	81	1.98%	172	4.21%	837	20.49%
Oct 01, 2022	3977	76	1.91%	182	4.58%	794	19.96%
Jan 01, 2023	3858	85	2.20%	172	4.46%	769	19.93%
Apr 01, 2023	3942	88	2.23%	181	4.59%	744	18.87%
Jul 01, 2023	3926	103	2.62%	196	4.99%	753	19.18%
Oct 01, 2023	3810	114	2.99%	201	5.28%	757	19.87%
Jan 01, 2024	3781	124	3.28%	233	6.16%	755	19.97%
Apr 01, 2024	3843	123	3.20%	244	6.35%	769	20.01%
Jul 01, 2024	3878	124	3.20%	252	6.50%	802	20.68%
Oct 01, 2024	3906	143	3.66%	263	6.73%	828	21.20%
Jan 01, 2025	4010	163	4.06%	282	7.03%	860	21.45%

Table S2: Number of Durable LVAD Candidates with Status 2 and 3 under Status Escalation Policies

Date	Total Waitlist	6-8 Year Policy		5-7 Year Policy		2-4 Year Policy	
		N	%	N	%	N	%
Apr 01, 2025	4108	180	4.38%	289	7.04%	898	21.86%
Jun 01, 2025	4158	194	4.67%	301	7.24%	917	22.05%

Table S3: Number of Durable LVAD Candidates, Stratified by Duration of Device Support

Date	Total Waitlist	Total LVAD	6-8 Years (N)	<6 Years (N)	≥8 Years (N)	6-8 Years (%)	<6 Years (%)	≥8 Years (%)
Oct 18, 2018	5010	1644	24	1614	6	1.5	98.2	0.4
Jan 01, 2019	4895	1701	25	1664	12	1.5	97.8	0.7
Apr 01, 2019	4889	1718	31	1673	14	1.8	97.4	0.8

Table S3: Number of Durable LVAD Candidates, Stratified by Duration of Device Support

Date	Total Waitlist	Total LVAD	6-8 Years (N)	<6 Years (N)	≥8 Years (N)	6-8 Years (%)	<6 Years (%)	≥8 Years (%)
Jul 01, 2019	4862	1738	34	1686	18	2.0	97.0	1.0
Oct 01, 2019	4794	1690	39	1635	16	2.3	96.7	0.9
Jan 01, 2020	4782	1722	37	1667	18	2.1	96.8	1.0
Apr 01, 2020	4574	1663	43	1601	19	2.6	96.3	1.1
Jul 01, 2020	4220	1566	48	1505	13	3.1	96.1	0.8
Oct 01, 2020	4157	1526	56	1459	11	3.7	95.6	0.7
Jan 01, 2021	4139	1500	54	1433	13	3.6	95.5	0.9
Apr 01, 2021	4213	1454	52	1388	14	3.6	95.5	1.0
Jul 01, 2021	4270	1457	56	1386	15	3.8	95.1	1.0
Oct 01, 2021	4170	1379	66	1298	15	4.8	94.1	1.1
Jan 01, 2022	4143	1363	69	1281	13	5.1	94.0	1.0
Apr 01, 2022	4084	1342	72	1249	21	5.4	93.1	1.6
Jul 01, 2022	4084	1361	69	1273	19	5.1	93.5	1.4
Oct 01, 2022	3977	1306	60	1223	23	4.6	93.6	1.8
Jan 01, 2023	3858	1235	68	1140	27	5.5	92.3	2.2
Apr 01, 2023	3942	1240	77	1136	27	6.2	91.6	2.2
Jul 01, 2023	3926	1202	80	1088	34	6.7	90.5	2.8
Oct 01, 2023	3810	1184	97	1056	31	8.2	89.2	2.6
Jan 01, 2024	3781	1198	102	1063	33	8.5	88.7	2.8
Apr 01, 2024	3843	1197	108	1054	35	9.0	88.1	2.9
Jul 01, 2024	3878	1245	108	1097	40	8.7	88.1	3.2
Oct 01, 2024	3906	1269	119	1105	45	9.4	87.1	3.5
Jan 01, 2025	4010	1296	138	1109	49	10.6	85.6	3.8
Apr 01, 2025	4108	1308	151	1104	53	11.5	84.4	4.1
Jun 01, 2025	4158	1335	163	1116	56	12.2	83.6	4.2
